# Measuring longitudinal cognition: Individual tests versus composites

**DOI:** 10.1016/j.dadm.2018.11.006

**Published:** 2019-01-11

**Authors:** Erin M. Jonaitis, Rebecca L. Koscik, Lindsay R. Clark, Yue Ma, Tobey J. Betthauser, Sara E. Berman, Samantha L. Allison, Kimberly D. Mueller, Bruce P. Hermann, Carol A. Van Hulle, Bradley T. Christian, Barbara B. Bendlin, Kaj Blennow, Henrik Zetterberg, Cynthia M. Carlsson, Sanjay Asthana, Sterling C. Johnson

**Affiliations:** a Wisconsin Alzheimer's Institute, University of Wisconsin School of Medicine and Public Health, Madison, WI, USA; b Geriatric Research Education and Clinical Center, William S. Middleton Memorial Veterans Hospital, Madison WI, USA; c Wisconsin Alzheimer's Disease Research Center, University of Wisconsin-Madison School of Medicine and Public Health, Madison, WI, USA; d Department of Communication Sciences and Disorders, University of Wisconsin, Madison, WI, USA; e Department of Neurology, University of Wisconsin School of Medicine and Public Health, Madison, WI, USA; f Department of Medical Physics, University of Wisconsin-Madison School of Medicine and Public Health, Madison, WI, USA; g Department of Psychiatry, University of Wisconsin-Madison School of Medicine and Public Health, Madison, WI, USA; h Department of Psychiatry and Neurochemistry, Institute of Neuroscience and Physiology, the Sahlgrenska Academy at the University of Gothenburg, Mölndal, Sweden; i Clinical Neurochemistry Laboratory, Sahlgrenska University Hospital, Mölndal, Sweden; j Department of Neurodegenerative Disease, UCL Institute of Neurology, Queen Square, London, UK; k UK Dementia Research Institute at UCL, London, UK

**Keywords:** Biostatistics, Longitudinal data analysis, Cognitive aging, Neuropsychological tests, Composite scores, Intraindividual variability

## Abstract

**Introduction:**

Longitudinal cohort studies of cognitive aging must confront several sources of within-person variability in scores. In this article, we compare several neuropsychological measures in terms of longitudinal error variance and relationships with biomarker-assessed brain amyloidosis (Aβ).

**Methods:**

Analyses used data from the Wisconsin Registry for Alzheimer's Prevention. We quantified within-person longitudinal variability and age-related trajectories for several global and domain-specific composites and their constituent scores. For a subset with cerebrospinal fluid or amyloid positron emission tomography measures, we examined how Aβ modified cognitive trajectories.

**Results:**

Global and theoretically derived composites exhibited lower intraindividual variability and stronger age × Aβ interactions than did empirically derived composites or raw scores from single tests. For example, the theoretical executive function outperformed other executive function scores on both metrics.

**Discussion:**

These results reinforce the need for careful selection of cognitive outcomes in study design, and support the emerging consensus favoring composites over single-test measures.

## Highlights

• Identifying early cognitive change requires tests with low error variance.
• In a middle-aged sample, composites were less noisy than single tests.
• Global and theory-driven composites outperformed data-driven composites.

## 1 Introduction

Understanding individual longitudinal cognitive change requires parsing multiple sources of variability in scores. In a longitudinal observational study, consistent decline may indicate true change, whereas a succession of rises and falls may not. However, true decline may be difficult to detect when changes are subtle and fluctuations over time are large—as in the beginning stages of a dementing disorder such as Alzheimer's disease (AD), where someone may meet criteria for mild cognitive impairment (MCI) at one visit but not the next [1]. Seeking measures with high test-retest reliability may not solve the problem, as the most stable tests may not be sensitive to early change. A more subtle criterion that directly assesses longitudinal variability is the intraindividual standard deviation (IISD) over repeated assessments [2]. Individuals with larger IISD may be at higher risk of subsequent dementia [1], [2], [3] or other impairment [4]; however, high IISD values in stable normal samples may be inflated by measurement error. Strategies for reducing error are necessary for understanding early cognitive decline.

To understand variability across tests and time, longitudinal studies of cognition typically include comprehensive cognitive batteries assessing many domains [5], [6]. Separate analysis of each outcome without considering familywise type I error risks spurious or irreproducible findings [7]. Alternatively, to reduce multiplicity, we can average individual tests into composite scores, as in, for example, the preclinical Alzheimer's cognitive composite (PACC), which combines scores from tests of memory and executive function [8]. Such composite scores have attracted attention as sensitive indicators of early cognitive change [9], and the FDA has indicated openness to cognitive composite endpoints for anti-AD drug trials [10]. Several approaches to devising composites have been proposed, including the data-driven approach, in which empirical data reduction techniques such as factor analysis are used to combine scores that tend to covary [11]; the theory-driven approach, in which established neuropsychological theories are used to combine scores within a single cognitive domain [12]; and the global approach, as in the PACC, in which representative tests from multiple domains are combined in a theory-driven way to estimate overall cognitive performance [8], [13]. In developing composites, reliability and validity must be considered in tandem, ensuring the composite reflects the construct of interest—a reduction in error variance must not come at the cost of a weakened relationship to the criterion [14]. If this is achieved, composite scores can limit type I error and reduce error variance, improving statistical power.

We assessed the suitability of several cognitive tests and composites for identifying cognitive change in the context of an ongoing longitudinal study of middle-aged and older adults. We aimed to (1) identify which measures have the lowest IISD, after adjusting for known sources of cognitive variability, and (2) examine the criterion validity of each measure by assessing its association with age and with amyloid-accelerated decline during late middle age.

## 2 Methods

### 2.1 Participants

Analyses used longitudinal neuropsychological data from participants in the Wisconsin Registry for Alzheimer's Prevention (WRAP), who are cognitively unimpaired at the baseline. Only visits with complete data were included. Participants having fewer than two complete visits (N = 397) or reporting a baseline neurological diagnosis (N = 43) were excluded. In addition, to ensure our measure of longitudinal inconsistency was not inflated by the presence of clinically significant decline, we also excluded participants who were diagnosed with MCI or dementia at any visit (N = 52). The effect of this exclusion criterion was examined in a sensitivity analysis (Section 2.4.5). After exclusions, this standardizing sample included data from 1063 participants with 2–5 visits (mean intervisit interval = 2.51 years). Participant characteristics are summarized in Table 1.

**Table 1:** Demographic characteristics of the WRAP sample

Sample characteristic	Cognitively unimpaired sample	Biomarker subsample	Excluded from standardization sample
N	1063	226	492
Age at WRAP recruitment, y, mean (SD)	53.9 (6.5)	54.8 (6.4)	54.9 (7)
Age at first visit selected, y, mean (SD)	58.2 (6.4)	58.7 (6.1)	–
Number of study visits included, median (range)	3 (2–5)	4 (2–5)	–
Sex, male, N (%)	322 (30%)	74 (33%)	137 (28%)
Education, some college or less, N (%)	399 (38%)	74 (33%)	252 (52%)
White/Caucasian	1014 (95%)	214 (95%)	360 (73%)
Black/African American	29 (3%)	8 (4%)	95 (19%)
Spanish/Hispanic	8 (1%)	1 (0%)	30 (6%)
American Indian/Native American	9 (1%)	2 (1%)	5 (1%)
Asian	3 (0%)	1 (0%)	1 (0%)
Parental history of AD, N (%)	772 (73%)	168 (74%)	357 (73%)
WRAT-3 reading standard score, median (range)	107 (66–120)	109 (66–119)	103 (45–120)
MMSE total, median (range)	30 (23–30)	30 (26–30)	30 (25–30)
Amyloid PET data, N (%)	–	206 (91%)	–
CSF amyloid data, N (%)	–	128 (57%)	–
Amyloid positive, N (%)	–	58 (26%)	–
Too few visits	–	–	397 (81%)
Baseline neuro dx	–	–	43 (9%)
Clin dx	–	–	52 (11%)

Abbreviations: AD, Alzheimer's disease; WRAP, Wisconsin Registry for Alzheimer's Prevention; PET, positron emission tomography; CSF, cerebrospinal fluid; MMSE, Mini-Mental State Exam; WRAT, Wide Range Achievement Test.

Full-sample validity analyses compared age effects across measures. Additional validity analyses used a subset with cerebrospinal fluid (CSF) and/or [^11^C]Pittsburgh compound B (PiB)-labeled positron emission tomography images, enabling in vivo estimates of amyloid burden (N = 226). To ensure the widest range of amyloidosis, this biomarker sample included 11 additional participants with MCI or dementia who had available amyloid estimates, but had been excluded from the standardizing sample. The effect of these participants on results was examined in a sensitivity analysis (Section 2.4.5).

Procedures were performed in compliance with ethical standards for human subjects research, and all participants provided informed consent.

### 2.2 Assessments

Participants in WRAP complete a comprehensive cognitive battery described in full elsewhere [5]. Cognitive tests incorporated in the current analyses include the Rey Auditory-Verbal Learning Test (AVLT) [15]; the Logical Memory subtest of the Wechsler Memory Scale—Revised (LM) [16]; the Brief Visuospatial Memory Test—Revised (BVMT) [17]; the Stroop test, Color–Word Interference (STROOP) [18]; the Trail Making Test, parts A and B (TMT-A and TMT-B) [19]; the Digit Symbol subtest of the Wechsler Adult Intelligence Scale—Revised (DIGSYM) [20]; the Controlled Oral Word Association Test, CFL version (CFL) [21]; and the Mini-Mental State Exam (MMSE) [22]. We quantified baseline literacy using the Reading subtest of the Wide Range Achievement Test—Third Edition [23].

### 2.3 Biomarker methods

Methods for processing CSF are described in full elsewhere [24]. Briefly, 22 mL of CSF were removed from the L3-L4 or L4-L5 vertebral interspace for each participant. These samples were processed at the Clinical Neurochemistry Laboratory at the Sahlgrenska Academy of the University of Gothenburg, Sweden. Samples were sent in batches at two time points and analyzed using commercially available enzyme-linked immunosorbent assay methods. CSF samples were assayed for Aβ_42_ and Aβ_40_ and corrected for batch as previously described [24]. 128 participants in the present study had available CSF Aβ_42_ and/or Aβ_40_.

206 participants underwent 70-minute dynamic [^11^C]PiB positron emission tomography scans (Siemens EXACT HR+) initiated with bolus injection (nominal 555 MBq). [^11^C]PiB radiochemical synthesis, positron emission tomography data acquisition, image processing and quantification have been described in depth previously [25]. The primary measure was average cortical [^11^C]PiB distribution volume ratio (reference Logan graphical analysis, cerebellum gray matter reference region, $\bar{k_{2}}$ = 0.149 min^−1^ [26], [27]) across eight bilateral regions of interest (angular, anterior, and posterior cingulate, medial orbitofrontal, supramarginal, middle, and superior temporal gyri, and precuneus) [28].

### 2.4 Statistical methods

#### 2.4.1 Composite measures

We considered five composites based on previous factor analyses of the WRAP battery [11], [29], representing immediate learning (EMP-IMM-LRN); delayed recall (EMP-DEL-REC); executive function (EMP-EXEC-FN); story recall (EMP-LM); and visuospatial learning (EMP-BVMT) (Table 2, columns 1–5). While item inclusion in the factor analysis was guided by theoretical perspectives on cognitive decline, the loadings and factor structure were data-driven; thus we refer to these as empirical composites (EMP). Although the cohort has grown since the first factor analysis, approximately 90 percent of the standardizing sample was in the earlier sample, and the baseline demographic characteristics of the overlapping samples were similar (Supplementary Table 1). Because some tests of interest were first administered at visit 2, the average age of sample members at the first visit included in these analyses is about 4 years older than the average baseline age reported elsewhere [5]. However, the factorial invariance by age noted in the original analysis justifies assuming that the factor structure remains a reasonable fit [11].

**Table 2:** Thirteen composite scores (columns) and the twelve raw test scores contributing to each (rows)

Raw scores	EMP-IMM-LRN	EMP-DEL-REC	EMP-LM	EMP-BVMT	EMP-EXEC-FN	THEO-IMM-LRN	THEO-DEL-REC	THEO-EXEC-FN	PACC4-MMSE	PACC3	PACC4-CFL	PACC4-TMTB	PACC3-TMTB
Rey AVLT Total	X∗	X∗	-	-	-	X	-	-	X	X	X	X	X
Rey AVLT Delayed	-	X	-	-	-	-	X	-	-	-	-	-	-
WMS-R Logical Memory-I	-	-	X	-	-	X	-	-	-	-	-	-	-
WMS-R Logical Memory-II	-	-	X	-	-	-	X	-	X	X	X	X	X
BVMT-R Total	-	-	-	X	-	X	-	-	-	-	-	-	-
BVMT-R Delayed	-	-	-	X	-	-	X	-	-	-	-	-	-
Stroop Color-Word	-	-	-	-	X	-	-	X	-	-	-	-	-
TMT Part A	-	-	-	-	X	-	-	-	-	-	-	-	-
TMT Part B	-	-	-	-	X	-	-	X	-	-	-	X	X
WAIS-R Digit Symbol	-	-	-	-	-	-	-	X	X	X	X	-	-
COWAT C,F,L	-	-	-	-	-	-	-	-	-	-	X	-	-
Mini-Mental State Exam	-	-	-	-	-	-	-	-	X	-	-	X	-

Abbreviations: AVLT, Auditory-Verbal Learning Test; BVMT-R, Brief Visuospatial Memory Test—Revised; COWAT, Controlled Oral Word Association Test; DEL-REC, delayed recall; EMP, empirical composites; EXEC-FN, executive function; IMM-LRN, immediate learning; LM, Logical Memory; MMSE, Mini-Mental State; PACC, preclinical Alzheimer's cognitive composite; THEO, theoretical composites; TMT, Trail Making Test; WMS-R, Wechsler Memory Scale–Revised.

NOTE. **X** in a cell indicates that the test represented in that row contributed to that column's composite. Empirical composite inputs (columns 1–5) were weighted according to the factor analysis on which they were based, as described by Koscik et al. [29]. Theoretical composites (columns 6–13) were computed using equal weights.

∗ Empirical factor analysis suggested alternate division of immediate and delayed portions of AVLT. EMP-IMM-LRN includes information from AVLT immediate trials 1 and 2; EMP-DEL-REC includes information from immediate trials 3–5 and delayed recall.

We also considered several theoretically derived composites (THEO). Three domain-specific theoretical composites, previously used in WRAP, represent immediate learning (THEO-IMM-LRN), delayed recall (THEO-DEL-REC), and executive function (THEO-EXEC-FN) [24] (Table 2, columns 6–8). We also considered five global composites (Table 2, columns 9–13), including the global preclinical Alzheimer's composite (PACC4-MMSE) [8]; a three-test PACC version omitting MMSE, due to its limited sensitivity in middle-aged healthy samples (PACC3) [30]; and a PACC version replacing MMSE with the CFL (PACC4-CFL) [31]. Furthermore, because one PACC test, DIGSYM, is not available in the National Alzheimer's Coordinating Center Uniform Data Set, Third Edition [6], we included two experimental versions of the PACC4 substituting TMT-B for DIGSYM, both with (PACC4-TMTB) and without (PACC3-TMTB) MMSE. Finally, we considered individual tests contributing to each composite.

To compute composites, we first standardized all scores (mean = 0, SD = 1). Where lower scores indicated better performance (TMT-A, TMT-B), scores were multiplied by −1. Each composite was created as an average of selected standardized raw scores (Table 2), with weighting scheme varying by composite type. Empirical composite inputs were weighted according to the factor analysis on which they were based, as described by Koscik et al. [29]. Domain-specific and global composites were unweighted averages of their components. All composites were then restandardized to a mean of 0 and a standard deviation of 1.

#### 2.4.2 Convergent and discriminant validity

We explored Spearman intercorrelations among all raw and composite scores. To explore the domain structure of the theoretical composites in a systematic way, we constructed a correlation matrix of constituent raw scores (similar to a multitrait-multimethod matrix [32]). Reliability estimates (diagonal) were calculated using intraclass correlation; between-outcome estimates (off-diagonal) were calculated using the repeated measures correlation, which adjusts for between-subjects performance differences [33], [34].

#### 2.4.3 Intraindividual longitudinal standard deviation

We estimated the longitudinal inconsistency of each outcome after factoring out known sources of variability. To do this, we constructed random-slopes models of each outcome, controlling for age, sex, education, literacy, and number of prior exposures to the battery, and output the residuals, such that the score for each variable at each person-visit represented the deviation from its predicted value given the covariates. For each subject and outcome, we then calculated the IISD of these residuals as a measure of inconsistency [35]. This provided a subjectwise estimate of the amount of longitudinal within-person variability not associated with known covariates.

#### 2.4.4 Criterion validity

Criterion validity was assessed by exploring relationships between each outcome, age, and (in the biomarker subsample) Aβ status. To examine age-related change across outcomes, we plotted 95% CIs of the ${\hat{\beta }}_{age}$ terms obtained from linear mixed models of each outcome controlling for covariates.

Primary subsample analyses treated Aβ as a binary variable, with 1 representing suprathreshold levels of PiB, CSF-Aβ_42_, or CSF-Aβ_42/40_, and 0 representing subthreshold values on each available marker. The processes for determining these thresholds for Aβ positivity have been reported in detail elsewhere [24], [36]. To estimate the proportion of variance attributable to Aβ_42_-related longitudinal decline, we regressed out covariate effects, and then modeled the residuals as a function of Aβ and Aβ× age. Next, we plotted the generalized *R*^2^ for these models ($R_{GLMM}^{2}$) [37]. To examine absolute effect sizes across outcomes, we plotted 95% CIs of the ${\hat{\beta }}_{A\beta \times age}$ terms obtained from linear mixed models of each outcome. Secondary validity analyses explored Spearman correlations between continuous Aβ biomarker values and individual age-slope estimates for each outcome.

#### 2.4.5 Sensitivity analyses

To examine the robustness of the IISD findings, we estimated mean IISD in a larger sample including 52 individuals that had previously been excluded due to a diagnosis of MCI or dementia during the study. We compared the average IISD for each outcome in this sample to the main findings and evaluated the differences in mean IISD between impaired and unimpaired individuals. In this expanded sample, we also compared IISD of all outcomes for a variety of risk groups to that observed in a lower-risk comparison group, as others have reported fluctuations in cognitive status in similar risk groups [4]. Parallel sensitivity analyses examined the robustness of the criterion validity findings to the removal of those with clinical impairment.

## 3 Results

### 3.1 Participants

Demographic information for the whole sample, the subset with CSF or PiB amyloid data, and the set who did not meet inclusion criteria are summarized in Table 1.

### 3.2 Convergent and discriminant validity

**Fig. 1:**
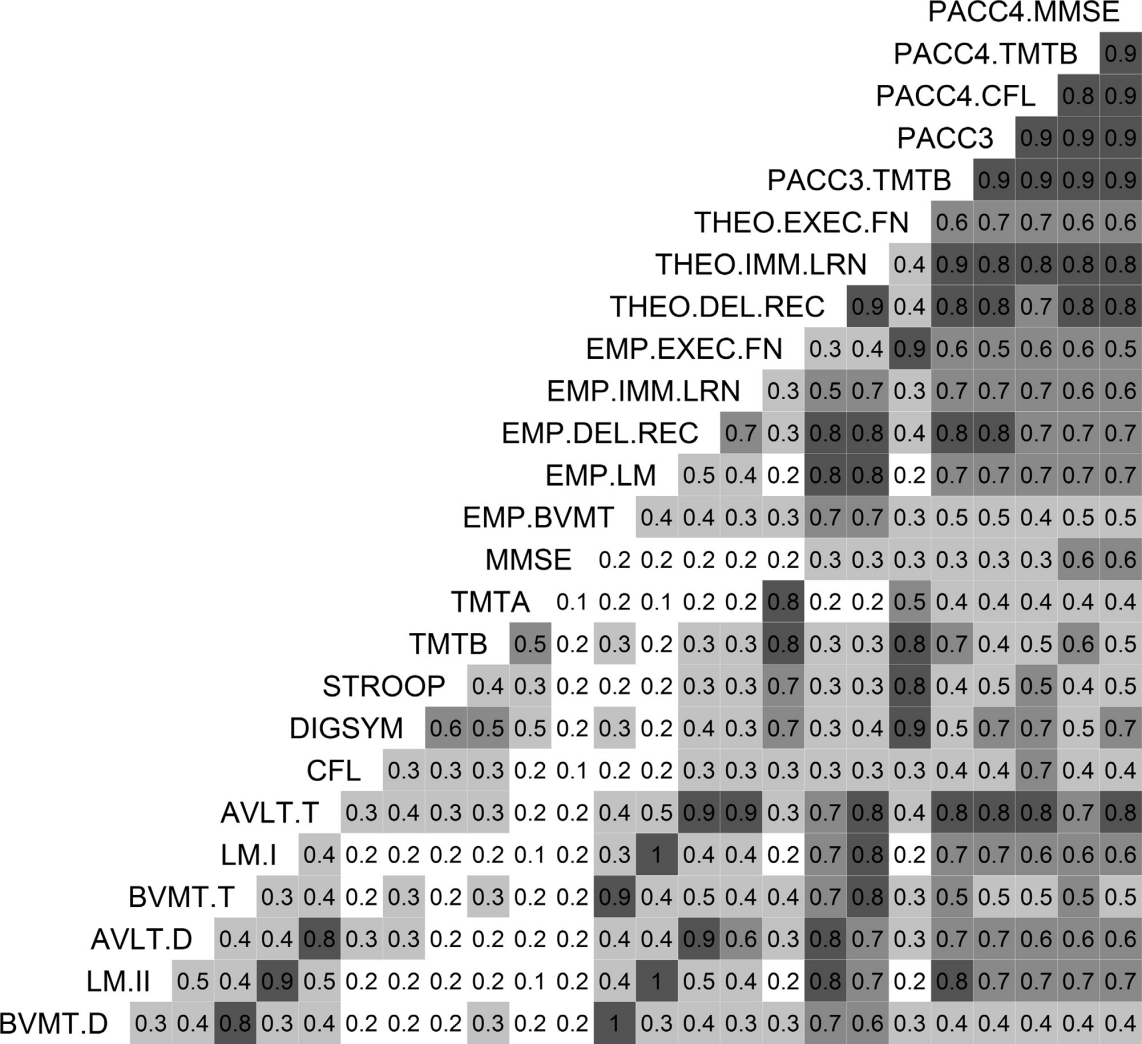
Correlogram illustrating relationships between all outcomes. Darker shading indicates correlations closer to 1. Abbreviations: AVLT, Auditory-Verbal Learning Test; BVMT-R, Brief Visuospatial Memory Test–Revised; DEL-REC, delayed recall; EMP, empirical composites; EXEC-FN, executive function; IMM-LRN, immediate learning; LM, Logical Memory; MMSE, Mini-Mental State Exam; PACC, preclinical Alzheimer's cognitive composite; THEO, theoretical composites; TMT, Trail Making Test; DIGSYM, Digit Symbol subtest of the Wechsler Memory Scale–Revised.

Intercorrelations among raw and composite scores are illustrated in Fig. 1. In general, scores related to executive function (STROOP, TMT-A, TMT-B, DIGSYM, THEO-EXEC-FN, EMP-EXEC-FN) were only weakly related to those in the episodic memory domains (AVLT-T, AVLT-D, LM-I, LM-II, BVMT-T, BVMT-D, THEO-IMM-LRN, THEO-DEL-REC, EMP-IMM-LRN, EMP-DEL-REC; median = 0.27, range = 0.07–0.41). Intercorrelations between memory-domain scores were stronger (median = 0.51, range = 0.27–0.97). Two raw scores in particular, MMSE and CFL, exhibited low correlations with all outcomes other than the related global composites (median = 0.24, range excluding related composites = 0.14–0.36). Intercorrelations were quite high among global composites (PACC4-MMSE, PACC4-CFL, PACC4-TMTB, PACC3, PACC3-TMTB; median = 0.9, range = 0.82–0.94) and between global and domain-specific composites (THEO-IMM-LRN, THEO-DEL-REC, THEO-EXEC-FN; median = 0.75, range = 0.62–0.86).

The matrix in Table 3 illustrates reliability and discriminant validity measures for three cognitive domains. Intraclass measures of reliability (within-domain, within-test) were reasonably high. However, the pattern of intercorrelations suggests a strong methods effect and relatively weak discriminant validity for the two memory domains. For executive function, within-domain, between-test correlations were similarly low, in line with other reports of high dispersion among executive function measures [38].

**Table 3:** Multitrait, multimethod matrix [32] evaluating the convergent and discriminant validity of the constructs represented by the immediate learning, delayed recall, and executive function theoretically derived composites

Raw scores	AVLT-T	AVLT-D	LM-I	LM-II	BVMT-T	BVMT-D	TMT-B	STROOP	DIGSYM
AVLT-T	***0.68***	**0.42**	*0.14*	0.15	*0.12*	0.07	0.03	0.05	0.06
AVLT-D		***0.68***	0.15	*0.17*	0.12	*0.05*	0.03	0.06	0.01
LM-I			***0.63***	**0.74**	*0.13*	0.08	0.04	0.07	0.09
LM-II				***0.68***	0.16	*0.11*	0.05	0.05	0.06
BVMT-T					***0.59***	**0.61**	0.08	−0.02	0.02
BVMT-D						***0.55***	0.06	0.01	0.03
TMT-B							***0.64***	*0.06*	*0.11*
STROOP								***0.82***	*0.22*
DIGSYM									***0.84***

Abbreviations: AVLT, Auditory-Verbal Learning Test; BVMT, Brief Visuospatial Memory Test–Revised; LM, Logical Memory; TMT, Trail Making Test; STROOP, Stroop test, Color–Word Interference; DIGSYM, Digit Symbol subtest of the Wechsler Adult Intelligence Scale–Revised.

NOTE. Main diagonal represents intraclass correlation (ICC) for within-subject variability. Off-diagonal represents repeated measures correlations between tests, adjusting for subject-level variance. Cells denoting pairwise comparisons within a test are bolded; cells denoting comparisons within a domain are italicized.

### 3.3 Intraindividual longitudinal variability

**Fig. 2:**
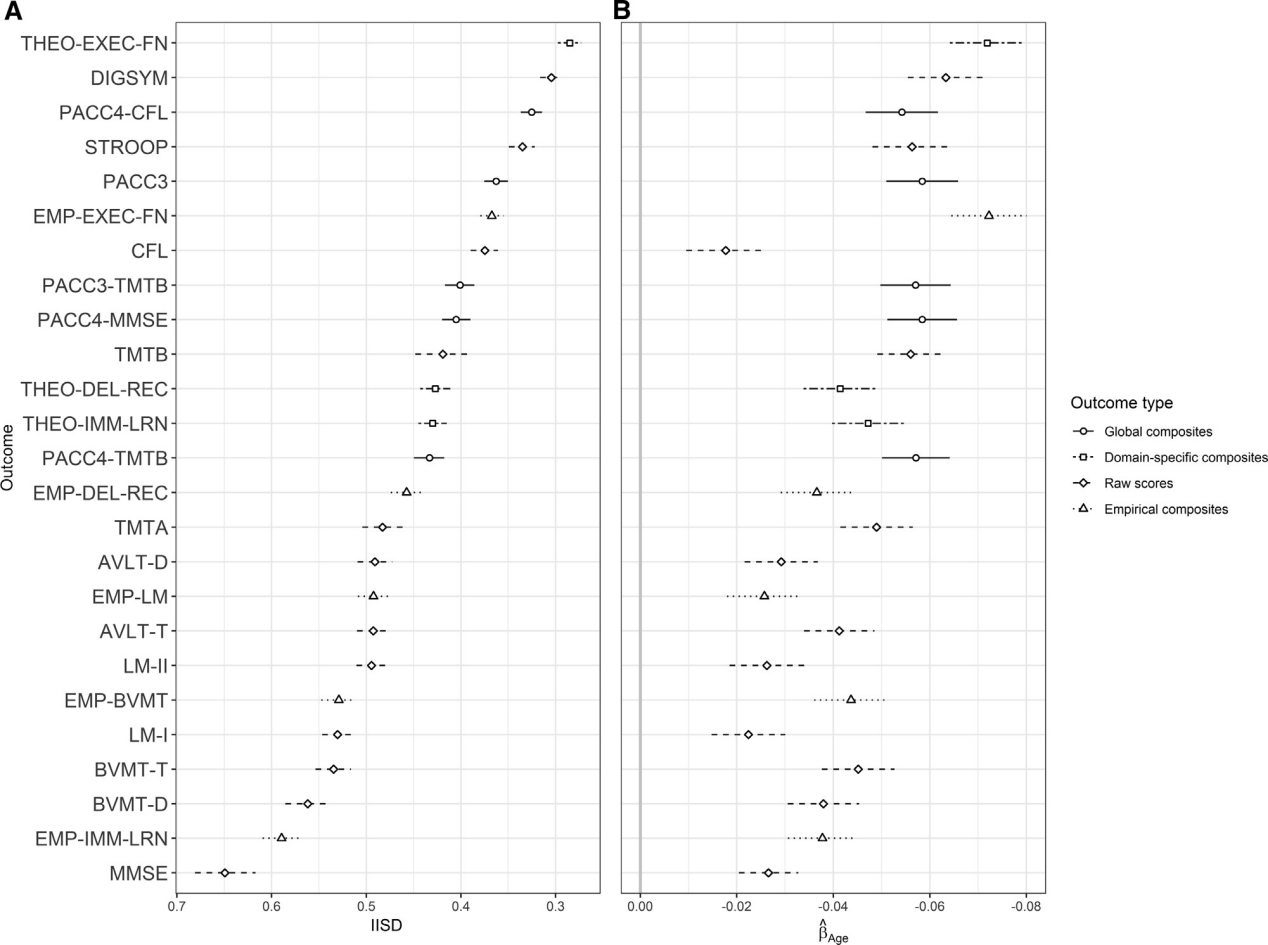
Performance of individual cognitive scores on two metrics of interest in entire sample (N = 1063). The y-axis is ordered by ascending mean IISD. Each x-axis has been oriented such that scores further to the right indicate more favorable measurement characteristics (A: lower IISD; B: greater sensitivity to age-related decline). (A) Mean intraindividual standard deviation (IISD) for all outcomes, with bootstrapped 95% confidence intervals. (B) Parameter estimate describing age-related change from full models of cognitive outcomes including other covariates (sex, education, baseline literacy, and prior practice with the battery). Error bars represent parametric 95% confidence intervals around the estimate. Abbreviations: AVLT, Auditory-Verbal Learning Test; BVMT-R, Brief Visuospatial Memory Test–Revised; DEL-REC, delayed recall; EMP, empirical composites; EXEC-FN, executive function; IMM-LRN, immediate learning; LM, Logical Memory; MMSE, Mini-Mental State Exam; PACC, preclinical Alzheimer's cognitive composite; THEO, theoretical composites; TMT, Trail Making Test; DIGSYM, Digit Symbol subtest of the Wechsler Memory Scale–Revised.

Fig. 2A illustrates intraindividual variability in each score over time, using the standardization sample of cognitively unimpaired individuals (N = 1063). Within domains, composites had lower IISDs than individual test raw scores. However, executive function raw and composite scores were less variable than scores from other domains, and some global composites as well. The MMSE raw score exhibited the largest IISD.

### 3.4 Criterion validity

Age-related slope estimates (Fig. 2B) for all outcomes were negative, indicating general decline with age. The two executive function composites (EMP-EXEC-FN and THEO-EXEC-FN), the DIGSYM raw score showed the most age-related change; slightly less was observed for the four global composites. The remaining composites and raw scores had slopes closer to zero.

**Fig. 3:**
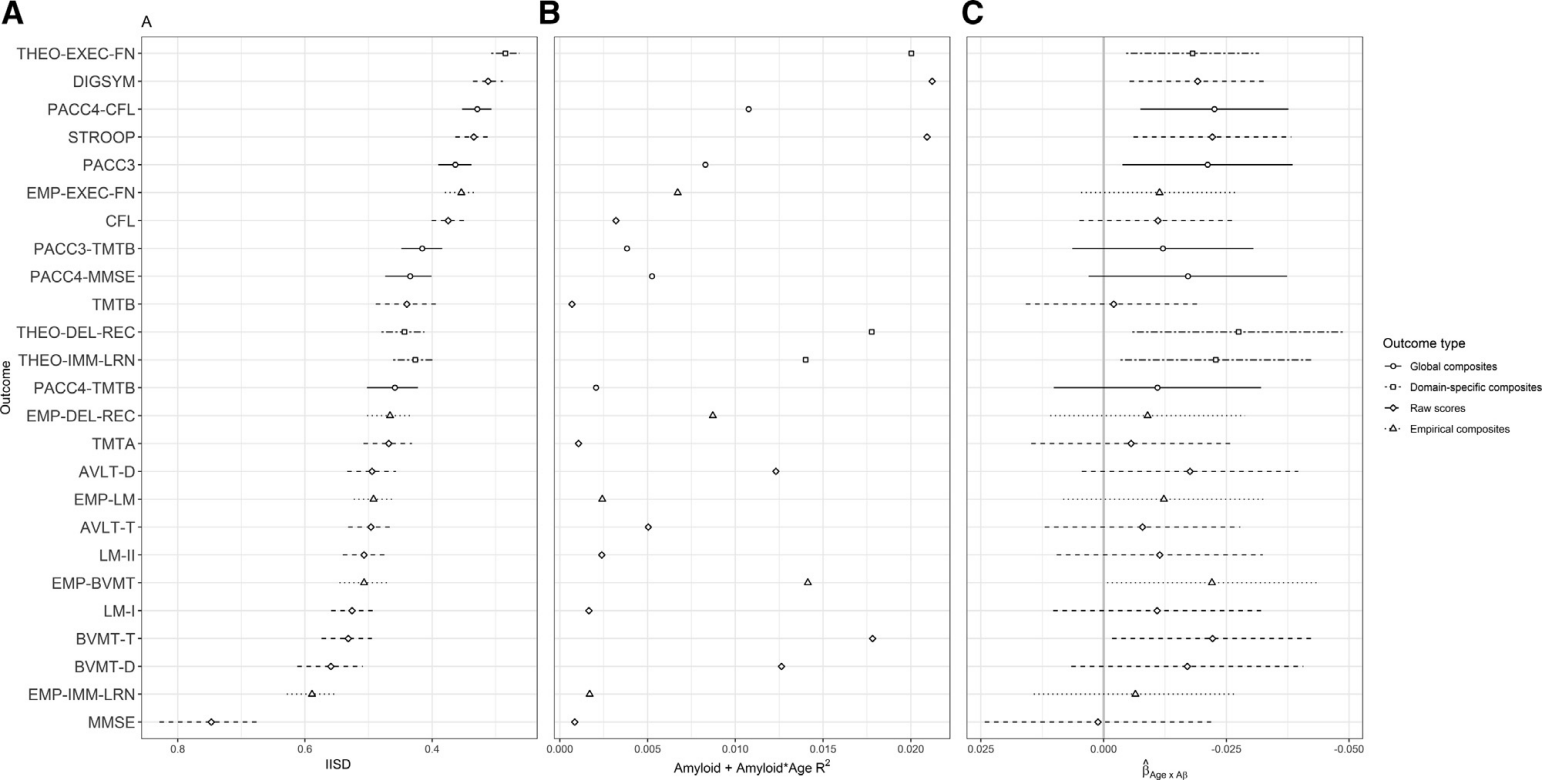
Performance of individual cognitive scores on three metrics of interest in the subsample having biomarkers (N = 226). The y-axis preserves the order of Fig. 2A. Each x-axis has been oriented such that scores further to the right indicate more favorable measurement characteristics (A: lower IISD; B-C: greater sensitivity to age-related decline). (A) Mean intraindividual standard deviation (IISD) for all outcomes, with bootstrapped 95% confidence intervals. (B) The proportion of variance ($R_{GLMM}^{2}$) [37] in cognitive outcomes attributable to Aβ and its interaction with age, after adjusting for standard covariates (age, sex, education, baseline literacy, and prior practice with the battery). (C) Parameter estimate describing age × Aβ interaction from full models of cognitive outcomes including covariates and Aβ. Larger negative values for this parameter estimate suggest worse age-related change in Aβ-positive individuals. Error bars represent parametric 95% confidence intervals around the estimate. Abbreviations: AVLT, Auditory-Verbal Learning Test; BVMT-R, Brief Visuospatial Memory Test–Revised; DEL-REC, delayed recall; EMP, empirical composites; EXEC-FN, executive function; IMM-LRN, immediate learning; LM, Logical Memory; MMSE, Mini-Mental State Exam; PACC, preclinical Alzheimer's cognitive composite; THEO, theoretical composites; TMT, Trail Making Test; DIGSYM, Digit Symbol subtest of the Wechsler Memory Scale–Revised.

The biomarker subsample (N = 226) showed a very similar IISD pattern (Fig. 3A). Fig. 3B–C illustrates two quantities related to criterion validity of each score. In few cases did the proportion of variance (generalized *R*^2^) attributable to Aβ positivity and its interaction with age exceed 0.02, indicating weak relationships between Aβ positivity, cognition, and cognitive change in this largely cognitively unimpaired sample (Fig. 3B). Parameter estimates for the Aβ positivity × age interaction (Fig. 3C) generally indicated worse age-related change in the Aβ-positive group, but group differences were modest, with most confidence intervals including zero. Confidence intervals were smallest for executive-function measures and larger for other raw scores and empirical composites. All theoretical composites had point estimates on the larger end, and most global composites performed similarly.

In exploratory analyses (Supplementary Fig. 1), Spearman correlations between individual random slope estimates and three continuous Aβ biomarkers were also most consistently visible for executive function measures ($\rho_{CSF-A\beta_{42},DIGSYM}$ = 0.26; $\rho_{CSF-A\beta_{42}/A\beta_{40},DIGSYM}$ = 0.24; $\rho_{CSF-A\beta_{42}/A\beta_{40},EMP-EXEC-FN}$ = 0.21; $\rho_{PiB-DVR,STROOP}$ = −0.23) The remaining correlations were smaller (−0.2<ρ<0.2) and/or confined to a single biomarker ($\rho_{CSF-A\beta_{42},EMP-LM}$ = 0.25; $\rho_{CSF-A\beta_{42},LM-I}$ = 0.22), although all were in the expected direction, with better scores associated with larger CSF-Aβ biomarker values and smaller [^11^C]PiB distribution volume ratio values.

### 3.5 Sensitivity analyses

We recalculated IISD on a larger data set including participants with at least one diagnosis of clinical MCI or worse at any point during the study (N = 1115). Mean IISDs in this supersample were very similar to the standardization sample (r = 0.997), indicating low sensitivity of our results to this exclusion criterion. However, IISD values tended to be higher for the added participants, with greater discrepancies for some outcomes (e.g., TMT-B, $\Delta_{IISD}$ = 0.57) than others (STROOP, $\Delta_{IISD}$ ≈ 0). Supplementary Fig. 2 illustrates the relationships between mean IISD in this sample and the group difference in IISDs between cognitively unimpaired participants and those with clinically significant cognitive impairment. The global composites tend to cluster in the quadrant with lower mean IISD and greater discrepancies between the clinical and nonclinical samples.

Supplementary Fig. 3 illustrates IISD for each outcome in a healthy subgroup (APOEε3/ε3 participants who were in good health at last visit and reported no clinical or psychiatric diagnosis at any point; Supplementary Fig. 3, top row) and several risk groups (APOEε4 carriers; those reporting a major psychiatric diagnosis; those reporting fair or poor health at last visit; and those receiving a clinical consensus diagnosis at any time). In our sample, those with clinical MCI or worse appeared to have slightly elevated IISD on some outcomes. In contrast to Sugarman [4], other subgroups showed variability similar to the healthy subgroup.

Sensitivity analyses for our criterion validity findings, in which clinically impaired individuals were removed from the biomarker subset, also showed little difference from the primary analyses, with high correlations between two estimates of IISD (0.997), generalized *R*^2^ (0.988), and β_Aβ×age_ (0.984).

## 4 Discussion

In a sample of over 1000 cognitively unimpaired late middle-aged adults, we observed that global and theoretically derived domain-specific composites generally exhibited lower variability and stronger relationships with age and Aβ compared with raw scores or to empirically derived composites [11], [29]. This is broadly consonant with other findings [8], [10]. Although the global composites excluding MMSE exhibited slightly smaller IISDs (Fig. 2, Fig. 3A) and stronger relationships with Aβ (Fig. 3B, C), these differences might not replicate in other samples. The key feature distinguishing global and theoretical composites from other scores is that these composites average across tests which load on distinct factors [11], [29]. Variability induced by poor performance on only one test from a given theoretical domain is reduced, allowing time trends to become more visible.

Others have reported associations between intraindividual variability and cognitive impairment [1], [2], [3] or other neuropsychiatric problems [4]. We therefore conducted primary analyses in a sample without clinically significant cognitive impairment to simplify the interpretation of variability. In follow-up analyses, we wondered whether those measures with low mean IISD values in a healthy sample would be sensitive enough to early change in those who are impaired. Indeed, in a sensitivity analysis on an expanded sample, mean IISD values for each outcome were quite similar, and some lower-IISD measures nevertheless evinced higher intraindividual variability in a subsample receiving a clinical diagnosis of MCI or worse during follow-up. However, no evidence of greater cognitive variability in other risk groups was observed.

The discriminant validity evidence for separate immediate learning and delayed recall factors in this data set is quite weak (Table 3). This was moderately surprising, as previous analyses in this sample suggested separate immediate and delayed memory components for the AVLT [29]. A reanalysis incorporating single-trial-level data for each memory test might more closely mirror the earlier result. However, given the high correlation observed between the two theoretical memory composites (Fig. 1), it may be worth considering a memory composite incorporating both immediate and delayed information.

The strong correlations among global composites are of practical importance for researchers wishing to compare results across studies, as variation across neuropsychological testing batteries is a common feature. These results confirm and extend the work of Donohue and colleagues to create a composite that can be used with modification in multiple cohorts [13]. The scientific community has recently acknowledged the importance of replication studies in neuropsychology [7]; thus, having a class of lower-inconsistency, high-criterion-validity composites that can be modified based on availability of inputs is beneficial.

The superiority of executive function measures on both consistency and some criterion validity measures was unexpected, as changes in memory are often thought to be the earliest cognitive signal associated with AD [9]. Some other reports suggest executive function changes in early AD [39], [40], and the relationship between lower executive function and biomarkers of brain amyloidosis has been observed before in this preclinical cohort [12]. However, we caution that some of what appears in this article to be a consistency advantage of executive function tests may be principally a function of normal aging [41], rather than disease-related processes, as outcomes that change more reliably with age will look superior by our inconsistency metric. The slight apparent advantage of executive function scores in relating to biomarkers (Fig. 3B; Supplementary Fig. 1) was not consistent across all metrics (Fig. 3C) [12] and should not be overinterpreted, except as evidence that such measures are appropriate to include in a comprehensive cognitive battery. We will re-examine this question directly once more of the WRAP cohort has reached a clinical endpoint.

### 4.1 Limitations

In these analyses, we did not perform formal hypothesis tests comparing composites to each other, and the confidence intervals we present (e.g., around beta estimates) have not been adjusted for multiple comparisons. We chose this approach because in a clinical trial setting, one or two outcomes would be selected as primary, so what researchers most need is not the proof that these outcomes are statistically distinguishable—they may not be—but instead, an understanding of the range of longitudinal variation and strength of relationship with criterion variables that they might expect for each, in samples similar to WRAP.

The tests covered by our analyses also did not span the entire range of cognitive function. In particular, confrontation naming, assessed in WRAP using the Boston Naming Test [42], was not considered. Previous analyses in this cohort have suggested there is not yet enough variability in this measure for it to be a meaningful differentiator [43]. Instead, we focused on measures that were components of one of several composites of interest to us, so that we could more easily make relevant comparisons.

### 4.2 Conclusion and future directions

These results reinforce the need for careful selection of cognitive outcomes when designing studies, and provide support for composite over raw scores because of lower longitudinal intraindividual variability and stronger relationships with AD biomarkers. Future work building on these findings will examine the relevance of this inconsistency measure to clinical trial planning.

**Research in Context**

1. Systematic review: We used PubMed to find articles discussing intraindividual variability and the construction of composite scores. Interest in composites in particular is growing and several key articles are cited, with special emphasis on the work by Donohue et al. describing the Preclinical Alzheimer's Cognitive Composite.
2. Interpretation: We used the longitudinal intraindividual standard deviation to quantify the variability of different scores in the same set of participants. Like other research groups using different metrics, we found composites to be advantageous.
3. Future directions: Assessing criterion validity in a middle-aged cohort is difficult because of the lack of true clinical endpoints. Future work should examine whether low-IISD measures like the selected composites are also good prognostic indicators of the eventual development of dementia.
