# Supplementary material for: Measuring longitudinal cognition: Individual tests versus composites
Source: Alzheimers Dement (Amst). 2019 Jan 11;11:74–84. doi: 10.1016/j.dadm.2018.11.006 (PMC6816509; doi:10.1016/j.dadm.2018.11.006)
Supplement: Supplementary Table 1 and Supplementary Figures 1–3 [file mmc1.doc]

###### Supplementary Table 1.

Comparison of baseline characteristics of participants in the present analysis versus the original analysis on which empirical factor scores are based.

| Dataset | Age at baseline | Age at first visit in dataset | First visit in dataset | Female, N(%) | Four-year college degree, N(%) | Non-white, N(%) | Parental FH of AD, N(%) |
| --- | --- | --- | --- | --- | --- | --- | --- |
| Current analysis | 53.9 (6.5) | 58.2 (6.4) | 2 (2-4) | 741 (70%) | 664 (62%) | 49 (5%) | 772 (73%) |
| Original factor analysis | 53.9 (6.6) | 53.9 (6.6) | 1 (1-1) | 856 (69%) | 756 (61%) | 25 (2%) | 908 (74%) |

**Supplementary Figure 1.**

Correlations between estimated individual cognitive trajectories on all cognitive outcomes (rows) and three A
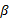
 biomarker outcomes (horizontal). Trajectories were operationalized as individual age slope estimates from linear mixed models relating age to each cognitive outcome, after adjusting for standard covariates (sex, education, and baseline literacy).

Left, highest global cortical [^11^C]PiB DVR score observed across all visits (
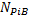
 = 206). Each participant was observed on up to 4 separate visits.

Middle and right, CSF-A
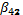
 and CSF-A
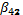
/A
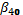
 ratio, respectively (
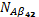
 = 128;
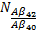
 = 127). Each participant provided CSF on one occasion.

**
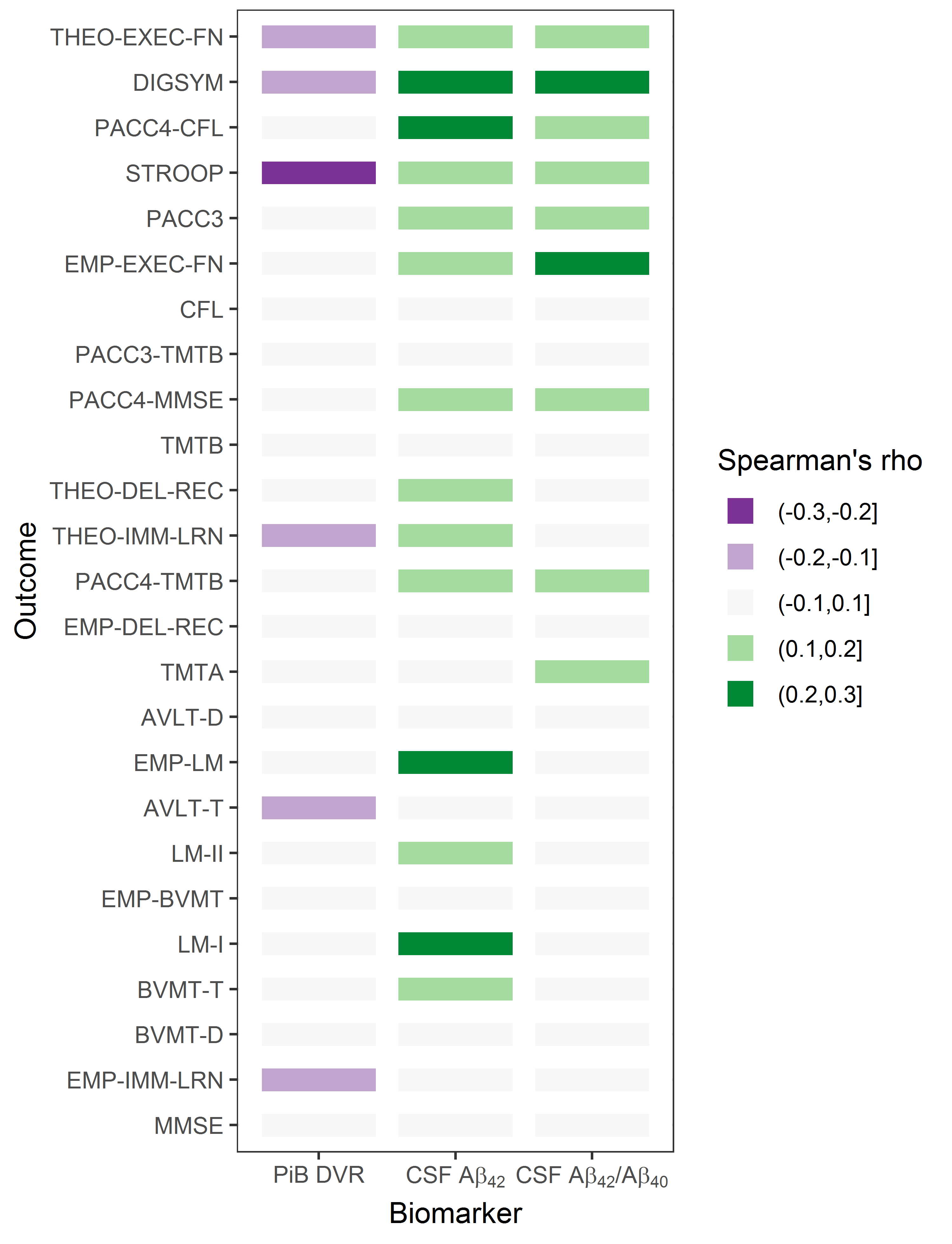
**

**Supplementary Figure 2.**

Relationships between average IISD (x-axis) and between-group differences (
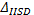
; clinical vs normal) for each outcome in the sensitivity sample (N=1115, including n=52 with clinical impairment). In keeping with Figures 2A and 3A, the x-axis has been oriented such that scores further to the right represent lower IISD.


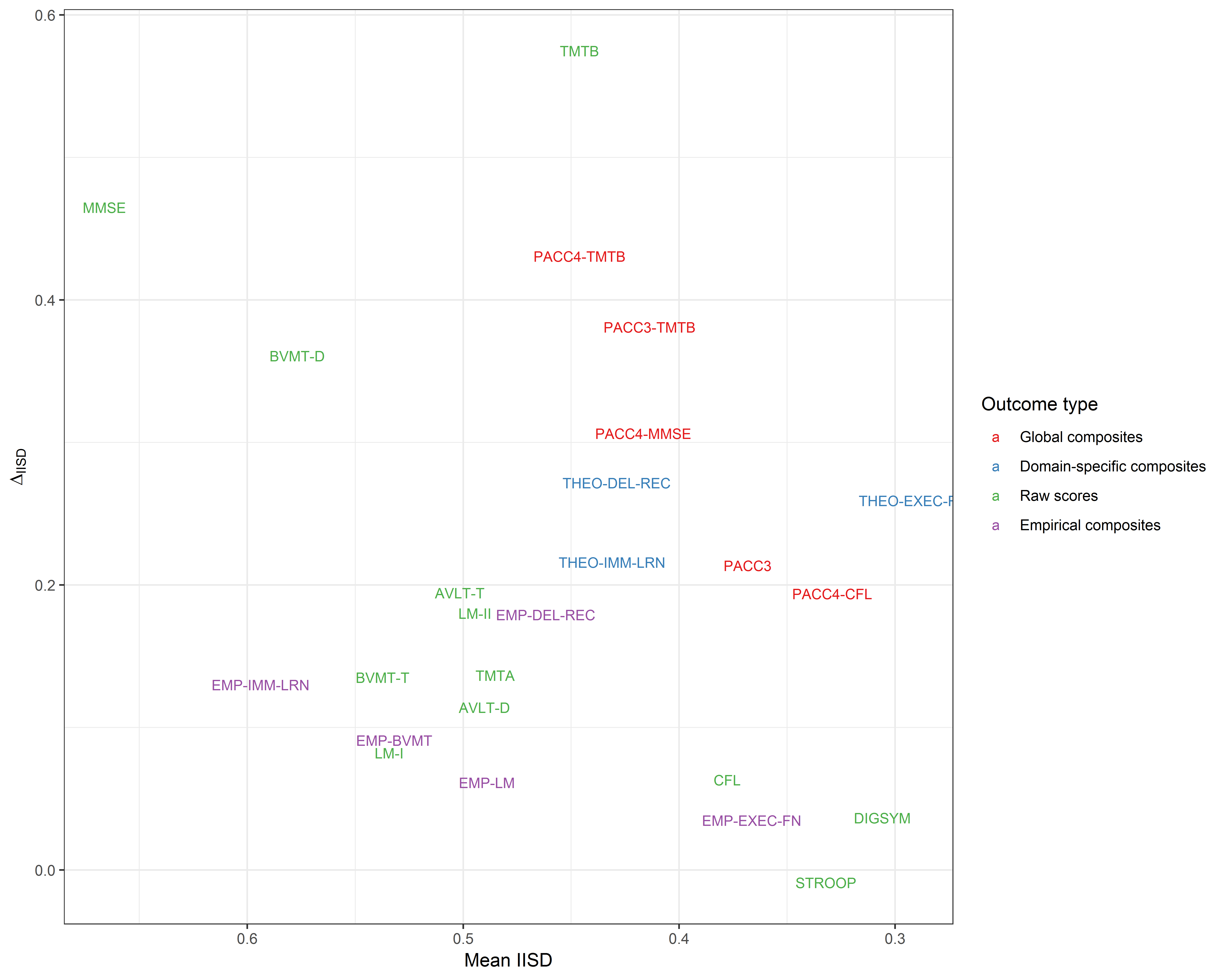


**Supplementary Figure 3.**

Comparison of IISD values (y) between healthy individuals and various other (non-exclusive) subgroups of interest, plotted separately for 25 outcomes. Individuals in the healthy group (top row of each subplot) were free of major cognitive and neuropsychiatric disorders for all visits, reported general health status of “Good” or better at last visit, and were homozygous for
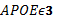
.
